# Supplementary material for: Gut microbiota profiles in feces and paired tumor and non-tumor tissues from Colorectal Cancer patients. Relationship to the Body Mass Index
Source: PLoS One. 2023 Oct 5;18(10):e0292551. doi: 10.1371/journal.pone.0292551 (PMC10553240; doi:10.1371/journal.pone.0292551)
Supplement: Attachment — Submitted filename: Response to Reviewers.docx [file pone.0292551.s001.docx]

**Response to the Reviewer≠1 questions:**

- CRC case-control implication has been removed (L70 in the previous version of the paper).
- A new paragraph has been started for the several parts of the primary results, adding informative headers, such as Reviewer suggested: lines 186-187 (NT/feces alpha diversity); lines 201-202 (NT/feces beta diversity); lines 219-220 (T/feces alpha diversity); lines 230-231 (T/feces beta diversity); lines 253-254 (T/NT alpha and beta diversity); lines 262-263 (tumor location, alpha and beta diversity); and lines 303-304 (alpha and beta diversity considering BMI values).
- In the Discussion section, the article recommended by Reviewer≠1 (doi: 10.1073/pnas.1406199111) has been considered: lines 396-402 and Reference number 24, in the revised version of the paper.
- At the end of the Discussion section (lines 430-433, we have included a paragraph addressing the limitations of our study. In this paragraph we consider, in addition to the heterogeneity in age and sex of the populations under study (comment included in the Conclusions section in the previous version of this paper), the limitations due to the number of subjects and the lack of statistical adjustment for multiple testing as mentioned by Reviewer≠1.

**Response to the Reviewer≠2 questions:**

- In the revised version of the manuscript, we have included a paragraph at the end of the Results section (lines 334-347) in which we summarize the most important data from our work. In addition, the Results section has been improved according to the recommendations of the Reviewer≠1. Specifically, a new paragraph has been started for the several parts of the primary results, adding informative headers. Finally, in the Conclusions section we have emphasized the important points of the manuscript.
- At the beginning of the Results section (lines 174-185), as Reviewer≠2 suggested, we have now better explained the results obtained from multiple LDA analyses with different levels of bacterial biological classification's. In order to facilitate the understanding of these data, we clarify that the level of LDA analysis that we showed depended on the number of taxonomic differences present in each particular comparison. Even though we are aware that the differences in bacterial genres may be more specific, when the differences were more abundant (e.g. in the comparison between feces and tumor or non-tumor tissue), we found more interesting to focus on the differences at the higher taxonomic levels (phyla, orders...) as they were more manageable and less confusing than the ones at lower taxonomic levels (LDA at the genus level were very big and difficult to include in a figure). However, when the differences were slighter (e.g. between tumor and non-tumor colorectal tissues), these did not reach the phyla or order levels, so in this case we included the LDA at the genus level with the purpose of refining the comparison.
- Alpha diversity Figures have been incorporated into the main Figures, and have been removed from the supplementary material.
- Data Availability Statement has been included. We have deposited the raw sequence data in a public repository:

Submission ID: SUB13573780
BioProject ID: PRJNA989099
<http://www.ncbi.nlm.nih.gov/bioproject/989099>
