# Supplementary material for: Gut microbiota profiles in feces and paired tumor and non-tumor tissues from Colorectal Cancer patients. Relationship to the Body Mass Index
Source: PLoS One. 2023 Oct 5;18(10):e0292551. doi: 10.1371/journal.pone.0292551 (PMC10553240; doi:10.1371/journal.pone.0292551)
Supplement: Attachment — Submitted filename: Response to Reviewers.docx [file pone.0292551.s002.docx]

**Response to the Reviewer≠1 questions:**

- Lines 174-185 from the previous version of the manuscript have been rearranged as Reviewer suggested **(lines 175-185 in the revised version)**.
- In the previous version of the manuscript, there was a typo since the sentence indicated by the Reviewer should not have been underlined. This typo has now been corrected **(lines 242-243 in the revised version)**.
- The sentence has been modified according to the Reviewer’s suggestion **(lines 262-263 in the revised version)**.

**Response to the Reviewer≠2 questions:**

- In the revised version of the manuscript, we have included new comments to clarify the relationship between obesity and CRC. Moreover, we have further discussed the potential implications in CRC of the observed differences in microbial composition between obese and normal with CRC patients in our study **(lines 452-456, 466-469, 472-484; and references 29, 30, 34, 35, 37, 38 39, in the revised version of the manuscript)**.

We consider that the modifications included in the Discussion section of the revised version of the manuscript contribute to better understanding the results of the work that are summarized in **lines 368-381**.

It is evident that it would be very interesting to carry out functional analyses in order to reach a complete understanding of the mechanisms involved in the results of the work. However, at this time it is not possible for us to do so. We continue working in order to obtain additional grants that would allow us to carry out functional studies in the future.
